# Combination therapy of atorvastatin and probucol on ischemic stroke in clinic

**DOI:** 10.1186/1479-5876-10-S2-A61

**Published:** 2012-10-17

**Authors:** Tao Yan, Feng-Rui Yang, Lin Meng

**Affiliations:** 1 Dept. Pharmacol. Tianjin Medical Univ. Tianjin 300070, China

## Background

Atorvastatin combined with probucol was considered in theory to be effects in treating ischemic stroke and preventing recurrent. However, the clinic data about it were very few, so we observed the effects of atorvastatin and probucol in combination in the patients with ischemic stroke.

## Methods

90 inpatients of emerging ischemic stroke were randomly selected in this study. All patients in research group,diagnosed by CT and MRI inspection, which comply with the diagnostic criteria of cerebral infarction amended by the fourth national conference. All of them signed informed consent document. And this study had unambiguous exclusion criteria. The patients were divided into 3 groups, atorvastatin + probucol group(n=30), atorvastatin group(n=30) , control group(n=30), observing for 6 months. Difference of every clinical index of these three groups was insignificant on admission. The blood lipids (including total cholesterol (TC), triglycerides (TG), low density lipoprotein cholesterol (LDL-C) and high-density lipoprotein cholesterol (HDL-C)), high-sensitivity C-reactive protein(hs-CRP) , pregnancy associated plasma protein (PAPP-A) level, intima-media thickness (IMT) and area of carotid intima of atherosclerotic plaque were tested before and after treatment respectively. The neurological deficit scores on the admission day and one month after admission were assessed, and the relationship between hs-CRP and the U.S. National Institutes of Health Stroke Scale(NIHSS)score and activities of daily living scale Barthel Index(BI) was analyzed.

## Results

A significant drop of blood lipids (P<0.01), IMT(P<0.01), area of Carotid intima of atherosclerotic plaque(P<0.01), PAPP-A(P<0.01) and hs-CRP(P<0.01) were observed in atorvastatin+ probucol group. All parameters were improved significantly comparing with atorvastatin alone. Neurological deficit scores of these two groups had significantly different after treatment (P<0.01). Combination therapy had better efficacy. The correlation analysis showed that seriousness of ischemic stroke on admission day and 1 month of treatment was associated with the hs-CRP levels significantly.

## Conclusion

Short-term use of atorvastatin combining with probucol could have a significant effect on ischemic stroke in clinic.

